# Rhizosphere wettability decreases with root age: a problem or a strategy to increase water uptake of young roots?

**DOI:** 10.3389/fpls.2013.00298

**Published:** 2013-08-13

**Authors:** Andrea Carminati

**Affiliations:** Department of Crop Sciences, Division of Soil Hydrology, Georg-August University of GöttingenGöttingen, Germany

**Keywords:** drying/wetting cycles, mucilage, neutron radiography, rhizosphere, root water uptake

## Abstract

As plant roots take up water and the soil dries, water depletion is expected to occur in the vicinity of roots, the so called *rhizosphere*. However, recent experiments showed that the rhizosphere of lupines was wetter than the bulk soil during the drying period. Surprisingly, the rhizosphere remained temporarily dry after irrigation. Such water dynamics in the rhizosphere can be explained by the drying/wetting dynamics of mucilage exuded by roots. The capacity of mucilage to hold large volumes of water at negative water potential may favor root water uptake. However, mucilage hydrophobicity after drying may temporarily limit the local water uptake after irrigation. The effects of such rhizosphere dynamics are not yet understood. In particular, it is not known how the rhizosphere dynamics vary along roots and as a function of soil water content. My hypothesis was that the rewetting rate of the rhizosphere is primarily function of root age. Neutron radiography was used to monitor how the rhizosphere water dynamics vary along the root systems of lupines during drying/wetting cycles of different duration. The radiographs showed a fast and almost immediate rewetting of the rhizosphere of the distal root segments, in contrast to a slow rewetting of the rhizosphere of the proximal segments. The rewetting rate of the rhizosphere was not function of the water content before irrigation, but it was function of time. It is concluded that rhizosphere hydrophobicity is not uniform along roots, but it covers only the older and proximal root segments, while the young root segments are hydraulically well-connected to the soil. I included these rhizosphere dynamics in a microscopic model of root water uptake. In the model, the relation between water content and water potential in the rhizosphere is not unique and it varies over time, and the rewetting rate of the rhizosphere decreases with time. The rhisosphere variability seems an optimal adaptation strategy to increase the water uptake of young root segments, which possibly reached new available water, and partly disconnect the old root segments from the already depleted soil.

## Introduction

Root water uptake depends on the relative importance of root and soil properties. When the soil is wet, rate and location of root water uptake are controlled by root traits. When the soil becomes dry, the soil hydraulic properties affect and, ultimately, limit the water availability to plant roots (Passioura, 1980; Draye et al., 2010).

In addition to the soil and plant hydraulic conductivity, the resistance of the root-soil interface has been supposed to affect root water uptake. Huck et al. (1970) and Carminati et al. (2009) showed that as roots take up water and the soil dries, roots shrink, and air-filled gaps form at the root-soil interface. Nobel and Cui (1992) estimated that in the intermediate dry range gaps are the limiting factor for root water uptake. In a classic paper, Passioura (1980) measured the total hydraulic conductance of soil and roots of an intact plant at controlled transpiration rates. After raising and decreasing the transpiration rate, he measured a decrease in total conductance of the system after the peak in transpiration. He interpreted this result as an increased resistance at the root-soil interface.

Increased resistance of the root-soil interface could be induced by root exudates. Hallett et al. (2003) measured a decrease in water sorptivity in the rhizosphere of barley, which is an indication of increased water repellency of the rhizosphere. Read et al. (2003) showed that lipids present in mucilage of maize, lupin and wheat decreased the surface tension of the soil solution, with a consequent reduction in the water holding capacity of the rhizosphere. Lipids may be responsible of the hydrophocity of the rhizosphere of lupins measured by Moradi et al. (2012).

It is well-accepted that mucilage favors root penetration by lowering the soil mechanical stress (Iijima et al., 2004). Instead, the direct effects of mucilage on root water uptake are still matter of debate. Carminati et al. (2010) observed higher water content in the rhizosphere than in the bulk soil during a drying period. The increase of water content in the rhizosphere was around 0.05 [cm^3^ cm^−3^]. Higher water content in the rhizosphere than in the bulk soil was observed also by Young (1995) for wheat. Young (1995) and Carminati et al. (2010) explained the higher water content in the rhizosphere with mucilage exudation. Indeed, McCully and Boyer (1997) showed that mucilage can hold large volume of water. However, they observed that mucilage lost most of the retained water at relatively high water potential. They concluded that mucilage should not play a major role in water storage in soils.

A successive experiment of Carminati et al. (2010) showed that after a cycle of drying and rewetting, the rhizosphere remained temporarily dry. Carminati (2012) hypothesized that the hysteretic and time-dependent behavior of the rhizosphere is explained by drying and wetting of mucilage exuded by roots. After drying, mucilage becomes hydrophobic and it rewets slowly. To include the specific behavior of mucilage, the Richards equation (the classic equation of water flow in soil) was modified by including a non-equilibrium term. It has not yet been proven that such a dynamic behavior of the rhizosphere is actually caused by mucilage.

Higher water content in the rhizosphere during drying and water repellency after rewetting have variable effects on root water uptake. A wet rhizosphere is expected to maintain the rhizosphere at a relatively high hydraulic conductivity also when the bulk soil dries. In fact, when the soil dries, large gradients in water potential are expected to occur in the rhizosphere. A rhizosphere with a high water holding capacity would attenuate this drop in water potential, facilitating root water uptake in dry soils (Carminati et al., 2011). On the other hand, the slow rewetting of the rhizosphere may limit root water uptake after drying and subsequent rewetting.

**Figure 1:**
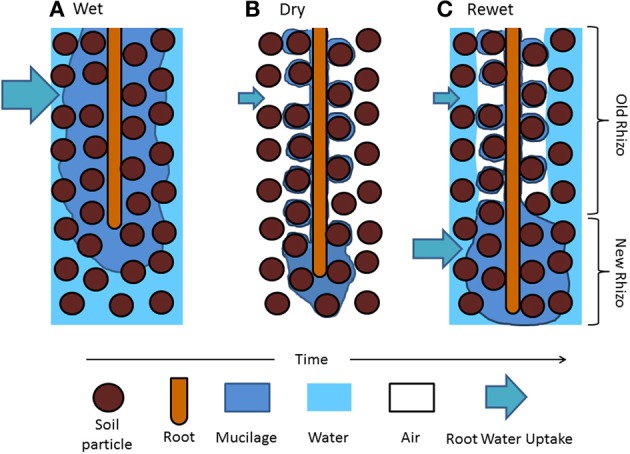
**Hypothetical distribution of mucilage and water in the rhizosphere during a drying and wetting cycle. (A)** Mucilage is exuded at the root tips and diffuses through the soil matrix. **(B)** As roots take up water and the soil becomes dry, mucilage dehydrates and shrinks around the root and the soil particle near the root (the *rhizosphere*). As the soil dries and mucilage becomes old, mucilage becomes water repellent and stiff. **(C)** After irrigation, old mucilage rewets slowly and the rhizosphere remains temporarily dry, possibly limiting the local water uptake. Freshly exuded mucilage covering the root tip is expected to rewet quickly. Water uptake might increase at the root apical segments.

This picture of water dynamics in the rhizosphere lacks important information: the variation of the rhizosphere properties along roots. Carminati and Vetterlein (2013) suggested that as roots grow, the rhizosphere at a given location becomes old and its hydraulic properties change. The authors suggested that young roots are covered with fresh and hydrated mucilage that helps the uptake of scares resources. Old roots are instead more isolated from the bulk soil because of gaps and/or water repellent and partly decomposed mucilage and are mainly responsible of long-distance transport. This concept is illustrated in Figure 1, in which the drying/wetting dynamics of the rhizosphere of a growing roots are simplified.

Objective of the present manuscript is to test the following hypotheses:

1. Does the rhizosphere of young roots rewet more quickly than the rhizosphere of old root? In other words: does the rewetting rate of the rhizosphere decrease with root age?
2. Is the rewetting rate of the rhizosphere function of the soil water content? In this case, is there a threshold water content below which the rhizosphere becomes hydrophobic?

To answer these questions we used neutron radiography to monitor the dynamics of water content in the rhizosphere of lupines during several drying/wetting cycles of variable length. The rhizosphere of young (distal) segments and that of older (proximal) segments were compared.

Finally, I implemented these results in the model of Carminati (2012). The model is modified to include temporal changes in the rewetting rate of the rhizosphere. This model is ready to be implemented in architectural models of root water uptake (Roose and Fowler, 2004; Doussan et al., 2006; Javaux et al., 2008; Schneider et al., 2010).

## Materials and methods

### Sample preparation

Six lupins (*Lupinus albus*) were grown in quasi-2D aluminum boxes filled with sandy soil. The aluminum boxes were 30 cm high, 15 cm large, and 1 cm thick. Sandy soil was collected from the catchment of Chicken Creek located near Cottbus, Germany. The soil (sieved to a particle sizes smaller than 2 mm) consisted of 92% sand, 5% silt, and 3% clay. The boxes were placed horizontally with one of the large side open and the sand was slowly and continuously poured into the aluminum box through a 2 mm sieve to achieve a uniform sand packing and minimize layering. The large side was then closed, the samples were turned vertically, and they were gently shaken to achieve a stable sand packing. The samples had holes at the bottom that allowed irrigation from the bottom. The samples were irrigated by slowly immersing the samples into a water reservoir until the water table reached 5 cm above the bottom of the sample. The capillary rise was enough to wet the sample till the soil surface.

Lupins were germinated on filter paper soaked with CaSo_4_. After 24 h they were planted into the soil at ~0.5 cm depth. During the first 7 days, the samples were daily irrigated from the bottom with tap water. During the second week, the samples were irrigated every second day with a nutrient solution composed of (in mM): K_2_SO_4_, 0.35; KCl, 0.1; KH_2_PO_4_, 0.1; Ca(NO_3_)_2_, 0.1; and MgSO_4_, 0.5; and (in μM): H_3_BO_3_, 10; MnSO_4_, 0.5; ZnSO_4_, 0.5; CuSO_4_, 0.2; (NH_4_)Mo_7_O_24_, 0.01; Fe-EDTA, 20. Plants have been grown for 20 days in a climate chamber with a daily light cycle of 16 h light: 8 h darkness, light intensity of 300 μmol m^2^ s^−1^, day/night temperature of 24°C/19°C, and relative humidity of 60%.

### Neutron radiography

Neutron radiography is an excellent method to image root and water distribution in soil samples thinner than 1–2 cm (Moradi et al., 2009). Neutron radiography consists in guiding a parallel neutron beam through a sample and detecting the intensity of the beam transmitted behind the sample. The transmitted beam is detected by a CCD camera and the information is converted into a digital image. The detected image carries the information on the thickness and composition of the sample.

Because of the high sensitivity of neutrons to hydrous materials, water is efficiently detected in neutron radiography. The relation between water content and neutron attenuation is given by:

(1) $$-\log\left(\frac{I(x,z,t)-dc}{I_{\text{dry}}(x,z)-dc}\right)=L_{w}(x,z,t)\;\Sigma_{w}$$

where *x, z* ate the space coordinates of the field of view, *t* is time, *I(x, z, t)* is the transmitted beam intensity, *I*_dry_*(x, z)* is the transmitted beam intensity when the sample is dry (only container and dry soil), *dc* is the dark current (signal when there is no beam), ∑_*w*_ [cm^−1^] is the neutron attenuation coefficient of water, and *L*_*w*_ is the thickness of water in the beam direction. *I*_dry_(*x, z*) was measured before the samples were irrigated and lupines planted.

In pixels where there are no roots, the volumetric soil water content, θ [−], is given by θ = *L*_*w*_/*L*_tot_, where *L*_*tot*_ is inner thickness of the sample in the beam direction. In pixels including roots, θ is the average of the water content in the root and in the soil in front and behind it (Carminati et al., 2010). The water content θ estimated from the radiographs was compared to that directly measured with a balance (the weight of the dry sample was known). The two values matched well and confirmed the image analysis already validated in Carminati et al. (2010).

Roots were segmented (technical word that describes the classification of pixels belonging to roots) using the algorithm *Roottracker2D* developed by Anders Kaestner and described in Menon et al. (2007). After root segmentation, the soil water content was calculated as a function of distance from the roots. The water content of the rhizosphere was calculated as the average water content in the first 1.5 mm near the roots. The image processing was identical to that described in more details in Carminati et al. (2010). To compare roots of different age, rhizosphere and bulk water contents were averaged in regions of size 5 cm by 5 cm, which included at least four roots of similar age. In specific, we calculated rhizosphere and bulk water contents in the upper 3–8 cm and in the lower 23–28 cm. The upper region contained roots that were approximately 5–10 days old at the beginning of the experiment. The roots in the lower regions were 1–4 days old.

Neutron radiography was performed at the NEUTRA facility at the Paul Scherrer Institute (PSI), Villigen, Switzerland. The field of view was 18.3 × 18.3 cm, with a pixel size of 0.0179 cm. Two radiographs of the upper and lower part of the samples were needed to image the whole sample. Exposure time was 30 s.

### Drying and wetting cycles

**Figure 2:**
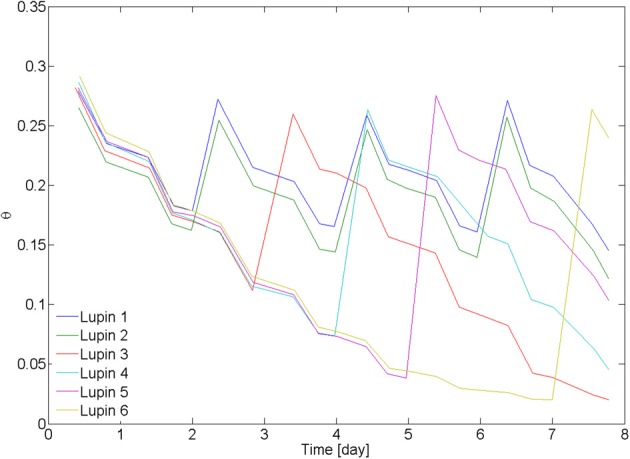
**Average water content in the samples.** Lupin 1 and 2 were irrigated from the bottom every 2nd day. Lupin 3–6 were irrigated once after a drying period of increasing duration. Water content was measured by weighing the samples. The decrease of water content became slower as the soil became dry, showing a decrease of transpiration. The samples were irrigated by capillary rise from the bottom, setting a water table at 5 cm above the bottom of the samples.

On day 20 after seed germination, the measurements with neutron radiography started. Two samples (L1 and L2) were irrigated every second day from the bottom. The other four samples were irrigated after 3 days (L3), 4 days (L4), 5 days (L6), and 7 days (L6). The average water content in the 6 samples, as measured from weighing the samples, is plotted in Figure 2. Time zero corresponds to the beginning of the neutron radiography experiment.

The samples were radiographed during day and night at intervals of 6 h. During irrigation, the samples were scanned before and 30 min after irrigation. The samples were weighed at the beginning and at the end of the photoperiod to measure the water consumption.

The neutron radiography experiment lasted 8 days.

### Model

Carminati (2012) proposed a new model that describes the changes in water content in the rhizosphere during a drying/wetting cycle. Because of the hydrophobicity of the rhizosphere after drying and its consequent slow rewetting, the water content in the rhizosphere, θ_rh_ [cm^3^ cm^−3^], does not increase as quickly as the soil matric potential *h*_rh_ [cm], here expressed in meter heads. In other words, although the matric potential increases, the rhizosphere remains dry for a long period. To describe this process, the assumption of a unique relation between θ_rh_ and *h*_rh_ has to be abandoned. Carminati (2012) suggested to describe the rewetting of the rhizosphere as:

(2) $$\frac{\partial \theta_{rh}}{\partial t}=C_{rh}\left(\theta_{rh}\right)\frac{\partial h_{rh}}{\partial t}+\Gamma_{rh}\left(\theta_{rh}\right)\;\left(h_{rh}-h_{rh}^{eq}\right)$$

where *C*_rh_(θ_rh_) [cm^−1^] is the specific soil capacity, *t* is time [s], Γ_rh_(θ_rh_) [m^−1^ s^−1^] is a function that describes the rewetting rate of the rhizosphere, and *h*^eq^_rh_ [cm] is the matric potential that the rhizosphere would have if it was in equilibrium—i.e., *h*^eq^_rh_ = *h*_rh_(θ_rh_).

In Carminati (2012), Γ_rh_ = Γ_rh_(θ_rh_) [m^−1^ s^−1^] was not function of time. Here I implement the model by considering that Γ_rh_ is also function of the root age. I define *t*_*i*_ [s] as the time when the root reached a given point in the space. The value of *t* − *t*_*i*_ [s] gives the age of the root at time *t*. In this way, I implicitly assume that mucilage is exuded only at the root tip. We parameterize Γ_rh_ = Γ_rh_(θ_rh_, *t, t*_*i*_) as:

(3) $$\Gamma_{\text{rh}}=\Gamma^{\text{sat}}\left(t\right)\Theta_{\text{rh}}{}^{\beta }$$

where $\Theta_{\text{rh}}=\frac{\theta_{\text{rh}}-\theta_{\text{rh}}^{\text{res}}}{\theta_{\text{rh}}^{\text{sat}}-\theta_{\text{rh}}^{\text{res}}}[{\text{cm}}^{3}{\text{cm}}^{-}{}^{3}]$ is the rhizosphere water saturation, θ^sat^_rh_ [cm^3^ cm^−3^] and θ^res^_rh_ [cm^3^ cm^−3^] are the residual and saturated water content of the rhizosphere, and Γ^sat^(*t*) [m^−1^ s^−1^] is the rewetting rate of mucilage at saturation which varies with root age according to:

(4) $$\Gamma^{sat}=\left(\Gamma_{rh}^{M}e^{-\gamma \left(t-t_{i}\right)}+\Gamma_{rh}^{m}\right)$$

where Γ^M^_rh_ [m^−1^ s^−1^] is the maximum rewetting rate of new mucilage and Γ^m^_rh_ [m^−1^ s^−1^] is the minimum rewetting rate of old mucilage. β [−] and γ [−] are two fitting parameters.

Equation (2) is combined with the Richards equation, the classical equation describing the water flow in soils:

(5) $$\frac{\partial \theta }{\partial t}=\frac{1}{r}\frac{\partial }{\partial r}\left[rk(h)\frac{\partial h}{\partial r}\right]$$

where *r* is the radial coordinate and *k*(*h*) is the soil unsaturated hydraulic conductivity [cm s^−1^]. Equation (4) is solved with an analytical approach under the *steady-rate approximation*, i.e., $\frac{\partial \theta }{\partial t}=\text{const}$ (Carminati, 2012). The solution is calculated in the two domains, bulk soil (*r*_1_ < *r* < *r*_2_) and rhizosphere (*r*_0_ < *r* < *r*_1_):

(6) $$\theta (r,t)=\{\begin{array}{c} \theta_{rh}(r,t)\;for\;r_{0}<r<r_{1}\\ \theta_{b}(r,t)\;for\;r_{1}<r<r_{2} \end{array}$$

(7) $$h(r,t)=\{\begin{array}{c} h_{rh}(r,t)\;for\;r_{0}<r<r_{1}\\ h_{b}(r,t)\;for\;r_{1}<r<r_{2} \end{array}$$

The radii of root, rhizosphere, and bulk soil were set equal to those used in Carminati et al. (2011): *r*_0_ = 0.05 cm, *r*_1_ = 0.25 cm, and *r*_2_ = 1 cm. The boundary conditions were no flux at *r*_2_ and constant flux at *r*_0_, *q*(*r*_0_) = −0.5 cm day^−1^. Initial condition was *h*(*r*_2_) = −20 cm. The water retention curves of rhizosphere and bulk were parameterized according to Brooks and Corey (1964):

(8) $$\Theta ={\left(h/h_{0}\right)}^{-\lambda }$$

(9) $$k=k^{\text{sat}}{\left(h/h_{0}\right)}^{-\tau }$$

where *k*^sat^ [cm s^−1^] is the saturated hydraulic conductivity, *h*_0_ is the air-entry value [cm], and λ [−] and τ [−] are fitting parameters.

The parameters for θ (*h*) and *k*(θ) of bulk soil and rhizosphere were taken from Carminati et al. (2011). The parameters were set to satisfy the following conditions: (1) at equilibrium, the rhizosphere is wetter than the bulk soil at any soil matric potentials. (2) The saturated hydraulic conductivity of the rhizosphere is 100 times smaller than that of the bulk soil. (3) At unsaturated conditions, the rhizosphere conductivity is higher than that of the bulk soil. The parameters for the rhizosphere rewetting [Equations (3, 4)] were estimated by matching the observed water content in the rhizosphere during the drying/wetting cycles.

## Results

The average water contents θ of the samples L1–6 during the drying/wetting cycles are plotted in Figure 2. The samples L1 and L2 were irrigated every second day and their average θ was between 0.15 and 0.3. L3 was irrigated at θ = 0.12, L4 at θ = 0.07, L5 at θ = 0.04, and L6 at θ = 0.02. After being rewetted, all samples reached the same water content of 0.26 ± 0.01. There was no visible effect of the drying/wetting cycles on the water repellency of the bulk soil. Transpiration rate started to decrease at ~θ = 0.05.

**Figure 3:**
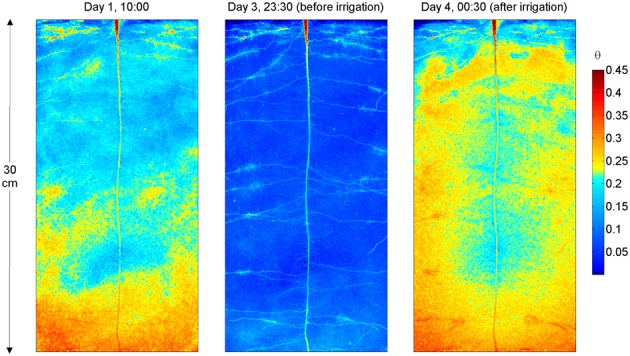
**Neutron radiography of L4, during the drying period and after irrigation.** The colormap is proportional to the water content. Note the higher water content near the upper roots at day 1. After irrigation (day 4), the rhizosphere of the upper roots, of the tap root, and that of the proximal parts of the lower roots remained markedly drier. On the other hand, the rhizosphere of the root tips in the lower parts of the sample rewetted and a region with high water content is visible around the root tips.

The water content distribution in L4 is shown in Figure 3. Figure 3 shows the images obtained after calibration of the neutron radiographs according to Equation (1) and after division by the neutron attenuation of water ∑_*w*_ and the sample thickness *L*_tot_. The images show the water content θ (*x, z*) and resulted from the superimposition of the neutron radiographs of the upper and lower halves of the sample. Figure 3 shows θ (*x, z*) during the drying period (day 1), just before irrigation (day 3 at 23:30), and 30 min after irrigation. On day 1 the rhizosphere of some of the upper roots, in particular in the vicinity of the cluster roots, appeared wetter than the bulk soil. On day 3 at 23:30, θ at the top of the sample is ~0.06 and it increased to 0.09 in the lower 5 cm of the sample. As the soil dried, roots became very well-visible. L4 had a long tap root that grew till bottom of the sample and laterals growing horizontally. The upper laterals showed several root clusters. All samples had a similar root architecture. The radiograph after irrigation shows that the rhizosphere of the upper lateral roots, of the tap root, and that of the proximal parts of the lower lateral roots remained markedly drier. Oppositely, the rhizosphere of the root tips in the lower parts of the sample quickly rewetted and a region with high water content appeared around the root tips.

**Figure 4:**
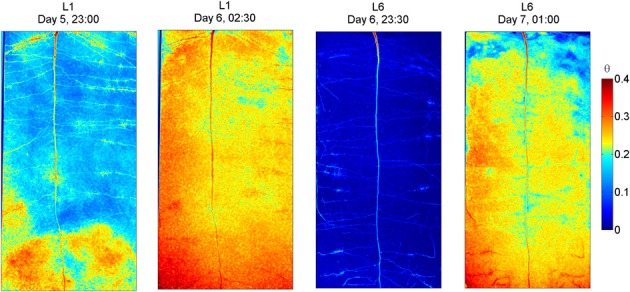
**Neutron radiography of L1 and L6 before and after irrigation.** L1 was irrigated every second day. Before irrigation its average water content was 0.17. After irrigation, the rhizosphere of the upper and middle part was not fully rewetted. Low and young roots were rewetted. L6 experienced a longer and more pronounced drying and was irrigated when its water content was 0.02 and the plant showed wilting symptoms. After irrigation the rhizosphere of most roots was not rewetted, except at the root tips, which appeared being covered with a blob of wet material.

The radiographs of L1 and L6 before and after irrigation are shown in Figure 4. As in L4, also in L1 some wet regions are visible around some of the lateral roots. After the 3rd wetting (day 6), the rhizosphere of the upper lateral roots of L1 remained drier than the bulk soil, indicating that a certain degree of water repellency occurred. The sample L6 was irrigated when the water content was uniformly low along the soil profile. After irrigation, the rhizosphere of the upper lateral roots, of the tap root, and of the proximal segments of the lower laterals remained dry, while the rhizosphere of the young segments of the lower laterals quickly rewetted. As in L4, a wet region appeared around the root tips of the lower laterals. These wet regions that I interpret as the wetting of freshly exuded mucilage, were larger in L6 than in L1.

To quantify the differences between the water content in the rhizosphere and in the bulk soil, I processed the images as in Carminati et al. (2010). Roots were segmented using the algorithm Roottracker2D (Menon et al., 2007). Then I calculated the water content as a function of distance to the root surface. This water content is still an average along the sample thickness—the radiographs are 2D, while the water distribution around roots is 3D. To calculate the actual average water content, I assumed that water content distribution around roots had a radial geometry and we fixed a rhizosphere extension of 1.5 mm. For more details see Carminati et al. (2010).

**Figure 5:**
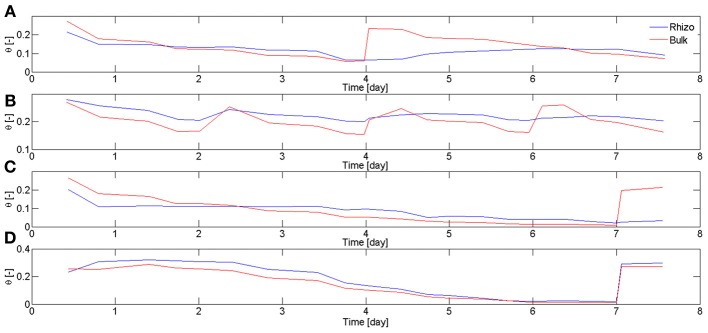
**Water content in bulk soil θ_*b*_ and rhizosphere θ_rh_ during drying and rewetting calculated for different samples and at different soil depth.** Time is calculated from the start of 1st drying cycle when the plants were 2 weeks old. **(A)** Average θ_*b*_ and θ_rh_ calculated in the top 5 cm of L4. During the drying period (day 2–4), the rhizosphere was wetter than the bulk soil. The situation reversed after irrigation, with the rhizosphere remaining temporarily dry. The rhizosphere slowly rewetted during the next 2 days. **(B)** θ_*b*_ and θ_rh_ at the top 5 cm of L1. The rhizosphere partly rewetted after the first irrigation. After the following irrigations the rhizosphere rewetted more slowly. **(C)** θ_*b*_ and θ_rh_ at the top 5 cm of L6. After sever drying and subsequent irrigation, the rhizosphere did not rewet as quickly as the bulk soil. **(D)** θ_*b*_ and θ_rh_ at the bottom 5 cm of L6, where only roots younger than 1 week were present (Figure 4). Although the bulk soil became very dry, the rhizosphere rewetted quickly and there was no sign of hydrophobicity. θ_rh_ was calculated for the most apical 4 cm of the roots.

In Carminati et al. (2010), the rhizosphere water content was averaged along the entire root system. Here, the rhizosphere water contents were averaged at different locations of the root system. In particular, we focused on two regions, the upper laterals and the lower laterals. The regions used for the calculation were ~5 × 5 cm and included around 5 roots. Figure 5 shows the average water content in bulk soil θ_*b*_ and rhizosphere θ_rh_ calculated for different samples and at different soil depth. Figure 5A, shows θ_*b*_ and θ_rh_ in the upper 5 cm of L4. During drying, θ_*b*_ decreased more rapidly than θ_rh_. Before irrigation, θ_rh_ > θ_*b*_. After irrigation, θ_rh_ increased much more slowly than the bulk soil. The average values of θ_*b*_ and θ_rh_ in the upper 5 cm of L1 are shown in Figure 5B. Figure 5B shows that the increase of θ_rh_ after rewetting became slower with the increasing number of cycles. The average values of θ_*b*_ and θ_rh_ in the upper and lower 5 cm of L6 are shown in Figures 5C,D, respectively. θ_rh_ in the upper region did not increase after rewetting. On the other hand, θ_rh_ increased very quickly in the lower region. The root segments in the lower region used for calculating θ_rh_ were ~5 days old at the time of rewetting, as estimated from the radiographs at the beginning of the experiment. Based on my previous experiments, I expect that the root segments in the upper region were approximately 2–3 weeks old. In fact, in lupines laterals in the upper region emerge approximately 1–2 weeks after planting.

## Discussion

Calculations of the water content in the rhizosphere are likely to be affected by root segmentation, by the contribution of root hairs and fine roots not resolved with neutron radiography, and by the image processing to go from the 2D pictures to the actual water contents in the rhizosphere. Therefore, some errors in the absolute values of the rhizosphere water content cannot be excluded. Instead, the relative difference between θ_*b*_ and θ_rh_ and its variation during the drying/wetting cycles are less prone to artifacts. In particular, the observed dryness of the rhizosphere compared to the bulk soil after rewetting and its slow rehydration are not affected by artifacts in the image analysis.

The experiments showed that:

1. Rhizosphere dynamics were not uniform along the root system. The rhizosphere rewetted slowly for the roots of the upper soil region and for the proximal segments of the roots of the lower soil region. On the other hand, the rhizosphere of the young segments of the roots in the lower soil region quickly rewetted. As the water content at the bottom was nearly as dry as at the bottom (Figures 5A,B), I conclude that the rewetting of the rhizosphere is related to the root age. In other words, young rhizosphere rewets quickly and old rhizosphere rewets slowly.
2. The slow rewetting of the rhizosphere occurred also at moist conditions (θ > 0.15, L1–2) and not only below a critical water content. Water repellency in the rhizosphere seems not to be function of the initial water content.

The slow rewetting of the rhizosphere after severe drying is caused by the high water repellency of the rhizosphere (Moradi et al., 2012). This water repellency is likely to be caused by mucilage exuded by roots. In fact, mucilage collected from lupine seeds and let dry on a thin glass turned hydrophobic (unpublished data). Such hydrophobicity can be caused by lipids present in mucilage (Read et al., 2003).

The radiographs of the samples after irrigation show a wet region around the root tips (Figures 3, 4). I interpret these wet regions as highly hydrated mucilage. This observation supports the results of McCully and Boyer (1997) that show that at high water potentials mucilage can hold large volumes of water and should appear as a blob around the root tips. The radiographs show also that freshly exuded mucilage rehydrates fast. On the other hand, mucilage that covers older root segments and that is likely to be as old as them, rehydrates more slowly. Rehydration time of mucilage seems therefore to increase with time. This increase can be the consequence of several factors. After that mucilage is exuded in soils, it reaches an equilibrium with the water potential in the soil. According to the relation between water content and water potential of mucilage measured by McCully and Boyer (1997), equilibration with a dry soil would cause a large dehydration of mucilage. During consequent shrinkage, mucilage is likely to become stiffer and therefore slower in rehydration. Additionally, as xylem vessels develop, root segments covered by mucilage become more and more active in root water uptake (Watt et al., 1994), which would cause additional suction and mucilage dehydration. Furthermore, interactions between mucilage and solutes present in the soil solution, ad example Ca^2+^, stabilize mucilage and make it stiffer (Carminati and Vetterlein, 2013). It cannot be excluded that microorganisms contribute to the mucilage stiffening as well.

Interaction between microorganisms and mucilage deserve a short discussion. Decomposition rates of mucilage-C by microorganisms may vary between 3 days (Nguyen et al., 2008) and 11 days (Mary et al., 1993). However, gel-like substances are not only decomposed but are also produced by microorganisms. Bacterial exopolysaccharides (EPS) has physical properties similar to those of mucilage (Chenu, 1993; Or et al., 2007). The mixture of plant derived mucilage and bacterial EPS is often called mucigel. It is commonly accepted that the rhizosphere properties are the result of plant-microorganisms interactions.

The fact that immediately after irrigation the rhizosphere does not rewet, suggests that the outer layer of mucilage in contact with the air-phase has a high water repellency, independently from the mucilage hydration state.

Another interesting observation is that the blob around the root tips after irrigation that I explain as mucilage, is larger in the samples that were exposed to drier conditions. If my hypothesis is right, this shows that mucilage exudation increases with drought stress. Mucilage can be a strategy of plants to increase the rhizosphere hydraulic conductivity when the soil dries, as proposed by Carminati et al. (2011).

**Figure 6:**
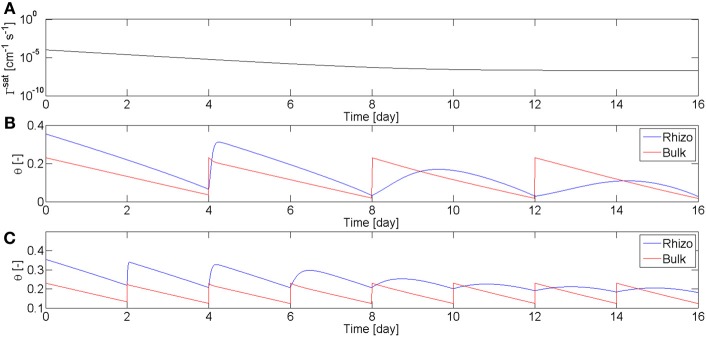
**Water content in rhizosphere (blue) and bulk soil (red) during drying and wetting cycles simulated using the model in Equation (2–5). (A)** Coefficient of wetting rate of water saturated mucilage, Γ^sat^, as a function of mucilage age according to Equation (4). **(B)** Simulation of the water content in rhizosphere and bulk soil during drying periods of 4 days and subsequent rewetting to a matric potential of *h* = −20 cm. During the first days, mucilage is fresh and rhizosphere is quickly rewetted. As mucilage ages, rhizosphere rewetting becomes slower. **(C)** Simulation with drying periods of 2 days.

These observations about the rewetting rate of mucilage are summarized in the model. Equation (3) means that the rewetting rate of the rhizosphere is a function of root age and water content. The dependence on the water content describes the fact that as mucilage dries it becomes stiffer and more viscous. I used these equations, coupled with the Richards equation in radial coordinates, to calculate the radial flow of water to a single root. Objective of the modeling was not to fit all the experimental data, but rather to find the parameters that qualitatively fit well with the overall results of the experiment. The function describing the dependence of Γ_rh_ on time, Equation (4), is plotted in Figure 6A. The parameter β describing the relation with the water content is set to β = 1.8. I simulated two scenarios, one in which the soil was rewetted every 4th day (as in L4), and one in which the soil was irrigated every 2nd day (as in L1–2). The calculated values of θ_*b*_ and θ_*rh*_ are plotted in Figures 6B,C. In the simulations the time corresponds to the root age. The parameters were chosen to match the experimental observations that the rewetting rates are fast for roots younger than 4–6 days, and are slow for roots older than 14 days. The model is capable of reproducing the general behavior of the observations.

**Figure 7:**
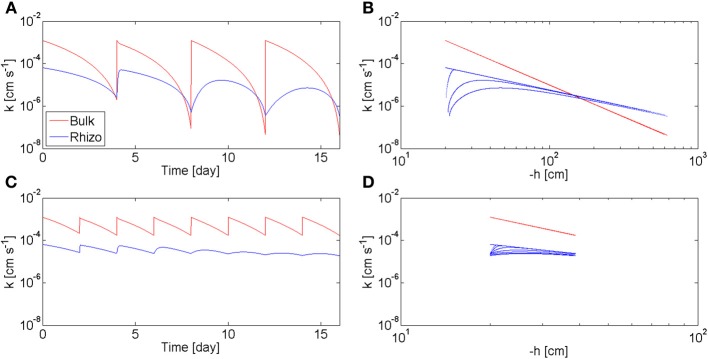
**Hydraulic conductivity of bulk soil *k*_*b*_ and rhizosphere *k*_rh_ during varying drying/wetting as predicted by the model. (A)** *k*_*b*_ and *k*_rh_ as a function of time during the 4 days drying cycles of Figure 6B. When the soil was wet, *k*_*b*_ > *k*_rh_. As the soil became dry, the two curves crossed each other and *k*_*b*_ < *k*_rh_. As the number of drying/wetting cycles increased, the recovery of *k*_rh_ after wetting became slower and slower. **(B)** *k*_*b*_ and *k*_rh_ as a function of the soil matric potential h during the 4 days drying cycles. **(C)** *k*_*b*_ and *k*_rh_ during to the 2 days drying cycles of Figure 6C. The soil was kept wet (θ_*b*_ > 0.1) and *k*_*b*_ > *k*_rh_ at all times. **(D)** *k*_*b*_ and *k*_rh_ as a function *h* during the 2 days drying cycles.

The model calculates also the values of the hydraulic conductivity of the bulk soil, *K*_*b*_, and of the rhizosphere, *K*_*rh*_, during the drying/wetting cycles. In Figure 7, *K*_*b*_ and *K*_*rh*_, calculated at the rhizosphere-bulk soil interface (*r* = *r*_1_), are plotted as a function of time (a, c) and as a function of the matric potential (b, d). The calculations are plotted for the long drying cycles (a, b) and for the short cycles (c, d). At the beginning of the long cycles, *K*_rh_ < *K*_*b*_. However, as the soil dried, *K*_*b*_ decreased more rapidly than *k*_*r*_ and there was a period before irrigation when *K*_rh_ > *K*_*b*_. During this period the rhizosphere favors root water uptake. Oppositely to *K*_*b*_, *K*_rh_ did not respond immediately to irrigation and its increase became slower with time. In the case of short cycles, the bulk soil remained always relatively wet (θ_*b*_ > 0.1), and *K*_rh_ < *K*_*b*_ during all time. The relation between hydraulic conductivities and soil matric potential is plotted in Figures 7B,D. In the bulk soil, the relation between *K*_*b*_ and *h* is a unique relation, i.e., *K*_*b*_ = *K*_*b*_(*h*) and at each *h* corresponds only one value of *K*_*b*_. This a classic situation in soil physics when no dynamics and no hysteresis are assumed. Instead, there is no unique relation between *k*_rh_ and *h*. The straight blue line in Figure 7B shows the values of *K*_rh_ at equilibrium. The figure shows that at equilibrium the hydraulic conductivity curves of rhizosphere and bulk soil crosses at *h* = −150 cm. During the 2nd, 3th, and 4th cycle, *K*_rh_ deviated from the equilibrium values. During the short-cycle scenario, deviation from equilibrium occurred after some cycles.

Figure 7 shows that rhizosphere is an advantage for root water uptake when the soil dries to matric potentials smaller than −150 cm. Below this matric potential, the rhizosphere is more conductive than the bulk soil and it is expected to favor root water uptake by limiting the drop in water potential toward the root (Carminati et al., 2011). Instead, when the soil remains relatively wet, as in the short-cycle scenario, the rhizosphere is less conductive than the bulk soil. Under this condition, the rhizosphere has no apparent advantages for root water uptake; actually, it may even be a limit to root water uptake. However, if we take a typical root hydraulic conductivity of 10^−7^ m s^−1^ MPa^−1^ (Draye et al., 2010) and we convert it into a rhizosphere hydraulic conductivity (assuming a rhizosphere of 1 mm thickness) we would obtain a conductivity of 10^−10^ cm s^−1^. The rhizosphere would be a limit of root water uptake only after severe drying, when the hydraulic conductivity decreased to 10^−10^ cm s^−1^. If rewetted after such a severe drying, the rhizosphere would remain dry for some period of time and it would be a limit to root water uptake.

These considerations indicate that, when the soil is relatively wet, plant roots have no reason to modify the rhizosphere properties in order to take up water more easily. Instead, when the soil becomes dry, mucilage exudation maintains the rhizosphere wet, facilitating root water uptake. However, mucilage exudation has a drawback, as it slows down the rhizosphere rewetting after sever cycles of drying. Mucilage exudation would then help the uptake of young, distal root segments covered with fresh mucilage, but over time it would limit the uptake of old, proximal root segments.

Of course, it has to be kept in mind that mucilage exudation has a carbon cost. It is likely that it is a short-term response to water shortage, while on the long-term, if water shortage persists, exudation will decrease. Beside the carbon costs, the pro and contra of mucilage exudation depend on the capacity of roots to uptake water from distal roots and transport it to the shoot. Possibly, the rhizosphere hydrophobicity of the old root segments would help to avoid water loss from roots to soil and it would be beneficial for water transport to the shoot. Hydraulic lift would be reduced by such rhizosphere dynamics.

The effects of mucilage dynamics on the overall soil-plant water relations are expected to be plant specific. The effects depend on several factors. I focus on two of them: (1) the amount of mucilage exuded and its chemical composition; and (2) on the root architecture. Mucilage exudation is function of plant species. Read and Gregory (1997) reported that maize seedlings produce more mucilage than lupines, and that mucilage produced by lupines rehydrates more slowly after drying. The slow rehydration of lupine mucilage is possible the reason of the slow rewetting of the rhizosphere shown in my study. Considering the tap-rooted architecture of lupines and the high axial conductivity of the tap root, we expect that water uptake in lupines after a drying and wetting cycle shifts to lower soil layers, where lateral roots are relatively younger and can compensate the reduced uptake from the upper soil layers (where roots have a temporarily hydrophobic rhizosphere). However, the situation may be different in plants with fibrous root systems, like maize and wheat, in which the capacity of taking up water from distal root segments may be limited by a low xylem conductivity. In this case, the slow rewetting of the rhizosphere of the old root segments, might be a limit for plant recovery after drying and subsequent irrigation.

However, to quantitatively describe the effects of such rhizosphere dynamics on the overall plant-soil water relations, the single-root model here introduced should be implemented into three-dimensional root water uptake models that explicitly account for root architecture (Roose and Fowler, 2004; Doussan et al., 2006; Javaux et al., 2008; Schneider et al., 2010).

### Conflict of interest statement

The author declares that the research was conducted in the absence of any commercial or financial relationships that could be construed as a potential conflict of interest.
